# Protocol for iSISTAQUIT: Implementation phase of the supporting indigenous smokers to assist quitting project

**DOI:** 10.1371/journal.pone.0274139

**Published:** 2022-11-09

**Authors:** Gillian Sandra Gould (Judean), Ratika Kumar, Nicole M. Ryan, Leah Stevenson, Christopher Oldmeadow, Gina La Hera Fuentes, Simon Deeming, Rebecca Hyland (Kamilaroi), Kym Yuke (Yugambeh), Faye McMillan (Wiradjuri), Brian Oldenburg, Marilyn J. Clarke (Worimi)

**Affiliations:** 1 Faculty of Health, Southern Cross University, Coffs Harbour Campus, Coffs Harbour, NSW, Australia; 2 College of Health, Medicine and Wellbeing, The University of Newcastle, Newcastle, NSW Australia; 3 Hunter Medical Research Institute, Newcastle, NSW, Australia; 4 Queensland Health, Brisbane, Queensland, Australia; 5 Faculty of Health, University of Technology Sydney, Sydney, NSW, Australia; 6 La Trobe University, Melbourne, Victoria, Australia; 7 Baker Heart and Diabetes Institute, Melbourne, Victoria, Australia; The University of Queensland School of Public Health, AUSTRALIA

## Abstract

**Introduction:**

About 44% of Aboriginal and/or Torres Strait Islander women smoke during pregnancy compared to 12% of their general population counterparts. Evidence-based quit smoking advice received from health care professionals (HCPs) can increase smoking cessation rates. However, HCPs lack culturally appropriate smoking cessation training, which is a major barrier to provision of smoking cessation care for this population.

**Methods and analysis:**

iSISTAQUIT is a multicentre, single arm study aiming to implement and evaluate the evidence-based, culturally competent iSISTAQUIT smoking cessation training among health practitioners who provide support and assistance to pregnant, Aboriginal and Torres Strait Islander women in Australia. This project will implement the iSISTAQUIT intervention in Aboriginal Medical Services and Mainstream Health Services. The proposed sample size is 10 of each of these services (total N = 20), however if the demand is higher, we will aim to accommodate up to 30 services for the training. Participating sites and their HCPs will have the option to choose one of the two iSISTAQUIT packages available: a) Evaluation- research package b) Training package (with or without continued professional development points). Training will be provided via an online eLearning platform that includes videos, text, interactive elements and a treatment manual. A social media campaign will be conducted from December 2021 to September 2022 to raise brand and issue awareness about smoking cessation for Aboriginal and Torres Strait Islander women in pregnancy. This national campaign will consist of systematic advertising and promotion of iSISTAQUIT and video messages through various social media platforms.

**Analysis:**

We will use the RE-AIM framework (Reach, Effectiveness, Adoption, Implementation and Maintenance) to plan, evaluate and report the intervention impact of iSISTAQUIT. Effectiveness of social media campaign will be assessed via social media metrics, cross-sectional surveys, and interviews.

**Discussion:**

This innovative research, using a multi-component intervention, aims to practically apply and integrate a highly translatable smoking cessation intervention in real-world primary care settings in Aboriginal Medical Services and Mainstream services. The research benefits Aboriginal women, babies and their family and community members through improved support for smoking cessation during pregnancy. The intervention is based on accepted Australian and international smoking cessation guidelines, developed and delivered in a culturally appropriate approach for Aboriginal communities.

## Introduction

Tobacco smoking (referred to as smoking from here-on) represents the most important preventable risk factor for chronic disease in Aboriginal and Torres Strait Islander People. About 44% of Aboriginal and/or Torres Strait Islander women smoke tobacco during pregnancy compared to 12% of their general population counterparts. Serious effects from smoking in pregnancy include complications during the pregnancy and perinatal period, as well as heart disease, obesity, and diabetes, and behavioural and learning problems in children later in life [1]. Maternal tobacco smoking is the most important preventable risk factor for chronic lung disease in offspring [2–6]. Babies born to women who smoke are twice as likely to have low birth weight compared to those born to non-smoking mothers [7], However, if the mother quits smoking early in pregnancy, the low birth weight risk decreases to non-smoking levels [7, 8]. A baby exposed to smoking in utero has more than a five-fold risk of early smoking initiation, thus continuing the cycle of disadvantage [9]. Abstinence by pregnant women can be an important cornerstone for the whole family’s respiratory health, by reducing tobacco smoking in the home, and by becoming community role models [10]. Nonetheless, Aboriginal and Torres Strait Islander pregnant women currently quit at only half the rate of their non-Indigenous counterparts [8, 11].

Interventions using counselling and/or NRT in Indigenous adult smokers have been found to be effective (RR1.43), [12] but have not successfully been implemented into the Indigenous context for pregnant women. Four tobacco-related randomised controlled trials (RCTs) have been conducted among Indigenous pregnant women who smoke in Australia [13] and Alaska, [14–16]. The trial in Australia, compared brief interventions with more intensive intervention [13]. The three Alaskan trials included a feasibility trial of a multi-component intervention [14],a biomarker educational program, [15] and a community-based support program with ‘native sisters’ [16]. None of the interventions were able to demonstrate efficacy of the intervention compared to control.

iSISTAQUIT seeks to translate into practice the considerable evidence that smoking cessation counselling from health providers (HCPs) helps smokers quit smoking, and has a focus on training of HCPs [17]. In an Australia-wide study that surveyed 378 GPs and obstetricians working in mainstream and/or Aboriginal and Torres Strait Islander settings, we found clinicians lacked confidence in prescribing nicotine replacement therapy (NRT) for both pregnant Aboriginal and Torres Strait Islander and non-Aboriginal and Torres Strait Islander women. Of these HCPs, 75% agreed that training would help improve their management of smoking in pregnancy [18]. Despite 95% believing that NRT is safer than smoking, only 5–21% prescribe NRT and only 5–14% follow-up women [19]. These rates are similar to studies conducted internationally [20, 21].

There are several cultural aspects for Aboriginal and Torres Strait Islander people, related to smoking that may be important for health providers to understand when helping people quit. These include the history of European colonisation and dispossession of land, the introduction of tobacco into Aboriginal and Torres Strait Islander communities, ongoing racism, intergenerational trauma and the social norms of tobacco use in everyday life [22]. Thus, it is important that a targeted smoking cessation program prepares the health professional to be sensitive to the cultural and psychosocial context of tobacco smoking and avoid shaming women for smoking in pregnancy [23]. While tobacco smoking is largely commercial in nature by Aboriginal and Torres Strait Islander people in Australia, there are some populations that may have ceremonial reference to tobacco, such as the Yolŋu people of East Arnhem land [24] and the use of a nicotine-containing plant called pituri, that is chewed [25].

Since lack of confidence and training in culturally sensitive smoking cessation methods may prevent health providers from delivering effective smoking cessation intervention, the iSISTAQUIT program was designed to use health provider training in smoking cessation as the primary intervention, accompanied by practical resources to assist the consultation process. Another salient feature of iSISTAQUIT is its social media campaign that was co-designed with Aboriginal and Torres Strait Islander women, community and health providers to increase the reach and adoption of the program. The combination of equipping HCPs to provide culturally safe smoking cessation care, and the use of social platforms, such as Facebook and the internet is supported by our previous research that suggests that a considerable proportion of Aboriginal women use internet and Apps for their and their children’s health related needs [26]. Internationally, a social media intervention for smoking cessation is being trialled in Alaska Natives [27].

The iSISTAQUIT program has been adapted from foundation work established by a pilot project called ICAN (Indigenous Counselling and Nicotine) QUIT in Pregnancy (Phase 2) that was developed and trialled in six Aboriginal Community-Controlled Health Organisations (ACCHOS) with the aim of training HCPs in culturally sensitive smoking cessation assistance to pregnant Aboriginal and Torres Strait Islander women who smoke, [28–30] as well as the SISTAQUIT Randomised Controlled Trial (Phase 3) [31]. This is intervention built on extensive formative research into pregnant Aboriginal and Torres Strait Islander women who smoke and health professional delivery of smoking cessation care [10, 32–40]. iSISTAQUIT is the implementation phase of SISTAQUIT and is based on same principles of smoking cessation training with additional adaptions including digital innovations in online training, medical practice software and a social media campaign.

## Material and methods

iSISTAQUIT is a quasi-experimental, [41] multicentre, single arm implementation study aiming to implement evidence based culturally competent iSISTAQUIT training among health practitioners that provide support and assistance to pregnant Aboriginal and Torres Strait Islander women in Australia.

### Objectives

This research has three main objectives:

1. Deliver the iSISTAQUIT training intervention in Aboriginal Community Controlled Health Organisations (ACCHO’s) and in mainstream health settings that service Aboriginal and Torres Strait Islander pregnant women.
2. Evaluate the delivery of iSISTAQUIT to services, considering process, impact and comparison between Aboriginal and Torres Strait Islander and Mainstream services
3. Develop, disseminate and evaluate a social marketing/social media campaign to motivate and aid pregnant women to quit and support health professionals in this work.

#### Ethics approvals

Ethics approvals have been obtained from Human Research Ethics Committees of University of Newcastle, Southern Cross University and Aboriginal Health and Medical Research Council. Where required, ethics approvals or ratifications have been obtained from respective states’ Aboriginal Health Research and Ethics Committee.

Current HREC approvals include:

### New South Wales

1. Southern Cross University HREC, Lismore NSW 2480 –Minimisation of Duplication Application Approval for iSISTAQUIT; Reference No. 2021/133. Approval date: 7/10/2021.
2. The University of Newcastle, Callaghan NSW 2308, Reference number H-2020-0162. Approval date: 12 May 2020
3. Aboriginal Health and Medical Research Council (AH&MRC) Strawberry Hills NSW 2012, Reference number 1140/15. Approval date: 23/04/2020

### Queensland

4. Far North Queensland HREC, Cairns & Hinterland Health Service District, Reference number HREC/18/QCH/27. Approval date: 2/4/2020
5. Darling Downs Hospital and Health Service (DDHHS): Reference number HREC/18/QTDD/10. Approval date: 09-04-2020

### Northern Territory

6. Top End HREC (NT Dept of Health/Menzies School of Health Research): Reference number 2017–2997. Approval date: 23-03-2020

### Western Australia

7. Western Australian Aboriginal Health Ethics Committee: Reference number 826. Approval date: 28-04-2020

### South Australia

Aboriginal Health Research Ethics Committee (AHREC) Aboriginal Health Council of South Australia. Reference number 04-16-652.

### Inclusion criteria and recruitment of sites

ACCHO’s will be recruited through their network. We will also invite participation from the Aboriginal Medical Services (AMSs) that have been interested in the SISTAQUIT RCT (Phase 3) [42] but were not eligible to join, or declined due to the research burden, or have finished being in the control arm of the SISTAQUIT RCT. We will also invite health services taking part in the Tackling Indigenous Smoking program, and other health services that express interest.

We will explore interest among Mainstream sites that see a high volume of Aboriginal and Torres Strait Islander pregnant women, to ensure we maximise our reach to Aboriginal and Torres Strait Islander women. We will also advertise in the media, through peak organisations and social media. We will opportunistically promote the recruitment of sites via conferences and specialised meetings. The scope of Mainstream services will include General Practice and Mainstream antenatal services (usually run by Local Health Districts or similar).

Consent will be obtained from the participating services in form of written, witnessed agreements. All health providers will provide consent in an electronic, online format. Service chief executive officers (CEOs) will provide written, informed and witnessed consent through paper consent forms for interviews. No minors will be involved in the study. All consenting health providers who see a pregnant woman in the participating services will be eligible to receive the iSISTAQUIT training and training resources.

Depending on COVID-19 restrictions, sites that express interest early in the recruitment period (early adopter services) will have an option to be involved in developing and filming the creative approach, campaign messages and video materials for the social media campaign.

#### Sample size

This project will implement the SISTAQUIT intervention in ACCHOs and Mainstream Health Services/sites. Sample size proposed is 10 of each health service or sites (N = 20), however if the demand is higher, we will aim to accommodate more services for the training, up to 30. With 20 sites, assuming an average of 8 HCPs trained per site are assessed for knowledge, attitudes and practices pre and post intervention, the study will have 90% power to detect a small to moderate standardised effect size of 0.2 standard deviations, with a 5% type 1 error rate.

### Intervention

Participating sites and their HCPs will have the option to choose one of the two iSISTAQUIT training packages available (Table 1).

**Table 1:** iSISTAQUIT package options.

Characteristics	Option 1: Evaluation-Research package (includes CPD points)	Option 2a: Training package with CPD points	Option 2b: Training Package without CPD points
iSISTAQUIT training	Yes	Yes	Yes
Research component	Yes	No	No
Surveys to complete	All surveys	Only pre- and immediate post-training HCP surveys acknowledged by HPMI as being done or not (irrespective of content) for CPD points	No surveys
Data collection	Medical practice software via Pen CS iSQ Topbar app (ABCD template) or Communicare iSQ clinical item (ABCD template)	None	None
Resources	Online training modules + treatment manual + patient flipchart + My journey booklet + NRT posters + ABCD desk mousepad + medical practice software templates + CO monitor/s + oral NRT	Online training modules + treatment manual + patient flipchart + My journey booklet	Online training modules + treatment manual + patient flipchart + My journey booklet
Option to get CPD points	Yes	Yes	No

ABCD: A-Ask/Assess, B- Brief Advice, C-Cessation, D- Discuss psychosocial context (of smoking)

CPD: Continued professional development

HPMI: Hunter Postgraduate Medical Institute

iSQ: iSISTAQUIT

#### Option 1. Evaluation research package

HCPs will be required to consent to completing the self-paced training modules, the reflective workbook and the pre- and post-training surveys (4 surveys in total). The training surveys will provide evidence on changes in knowledge, attitude and behaviours of the HCPs related to smoking cessation care. HCPs will be entered into a prize draw for completing all surveys and will have an option to claim CPD points.

The full iSISTAQUIT Evaluation-Research package consists, in addition to the training resources (treatment manual, patient flipchart and My Journey (patient) booklet), a) the provision of a carbon monoxide meter and oral nicotine replacement therapy (NRT), and, b) the choice of two medical practice software templates (Pen CS Topbar or Communicare) which aid and support the HCPs to enter the data about the patient’s medical consultation and the characteristics of the women’s quit attempts. The templates will be also utilised to guide the consultation according to the training using the ABCD approach [43]. ABCD approach stands for A—ask/assess; B—brief advice; C—cessation; and D—discuss the psychosocial context of smoking. This approach has been previously used in ICAN QUIT in Pregnancy and the SISTAQUIT RCT project.

#### Option 2. Training package

*Option 2a*. Training with professional or CPD points: HCPs complete the self-paced training modules and fill out the pre and immediate post training surveys. They also receive the treatment manual, patient’s flipchart and the My Journey (patient booklet). This option is suitable for HCPs that would like to do the training and obtain the professional or CPD points without participating in the research.

*Option 2b*. Training with no professional or CPD points: HCPs complete the self-paced training modules and receive the treatment manual, flipchart and My Journey patient booklet but do not complete the pre- and immediate post training surveys. This is for HCPs that do not want the professional or CPD points (for e.g. not a requirement in their profession) but would still like to get the benefits of the iSISTAQUIT training.

The two training schemes were introduced to cater to the capacity of the health services to be involved and the funder’s desire to increase services’ access to the program. Option 2 is offered to make the training equitably accessible to sites that otherwise would not be able to be involved in research due to the extra demands of the COVID-19 pandemic or other constraints.

For the rest of this paper, data collection and implementation of the study refers to services that choose option 1 (Evaluation-Research package), unless otherwise specified. Data from HCPs that choose option 2a will only be used for conferral of professional or CPD points and to calculate the program’s reach. Only data about reach will be collected from services that choose option 2b.

### Training delivery

Both training packages with the associated options will be available through the Moodle platform provided by the Hunter Postgraduate Medical Institute (HPMI). Moodle is a robust, secure, and integrated system learning platform for educators, administrators and learners. Moodle will be used for the associated training surveys (see https://moodle.com/security-privacy/) and participants will need to make an account to log into the HPMI website to access the online training and the surveys.

iSISTAQUIT training for both packages will have 14 e-Learning modules, taking approximately four hours to complete. The training will be internet-enabled and self-paced and includes videos, text, and interactive elements, supported by hardcopy resources i.e., the treatment manual and patient flipchart for HCPs and the My Journey (patient booklet), which includes augmented reality videos to aid smoking cessation care. The iSISTAQUIT e-Learning is designed to use the Eight Aboriginal Ways of Learning, is self-paced and accredited for professional development or CPD points with several professional colleges [44]. The Eight Aboriginal Ways of Learning involves using an Aboriginal pedagogy (such as story sharing, community links and symbols and images) and is congruent with the Australian Health Practitioner Regulation Agency (AHPRA) framework for cultural safe practice by HCP’s [44, 45].

### Site set-up

Consultations with and establishment of the Evaluation-Research sites will be completed by email, telephone and videoconference. The iSISTAQUIT Team will help facilitate the ethics processes and documentation required for the research collaboration. The team will also facilitate the provision of access to the electronic version of the iSISTAQUIT resources and to the e-Learning training via HPMI. Provision of hard copies of the iSISTAQUIT resources (patient booklets, flipcharts, treatment manuals), and provision of equipment (handheld Bedfont piCO Baby Carbon Monoxide Meter) and oral forms of NRT (with preference to those not currently subsidised on the Australian Pharmaceutical Benefit Scheme) will be facilitated by the team. Each service that chooses the full iSISTAQUIT Evaluation-Research package will be followed up by a site visit to support implementation, where feasible amid COVID19 restrictions. Geographic areas may be pragmatically selected for sequential roll out.

Services that opt for the iSISTAQUIT Training package, will similarly be established via the same communication channels, but will fill out a two-page registration form, giving details of their service and requirements. A communication log (CL) with the health services will be used to record the sites engaged, resources sent to them, potential participants, and information of the number of the annual number of pregnant women that reach the service.

#### Social media campaign

The research component of the social media campaign is being conducted from December 2021 to September 2022. After this date, the social media campaign may continue in a non-research capacity. The aims of the social media campaign are: to increase iSISTAQUIT brand recognition, increase awareness about smoking cessation for Aboriginal and Torres Strait Islander women in pregnancy, increase interest about the program and promote behaviour change among Aboriginal and Torres Strait Islander communities and health professionals. The creative approach for the social media campaign will be developed by the company Gilimbaa in partnership with IndigenousX who will be developing the social media strategy to deliver a comprehensive campaign. Gilimbaa and IndigenousX are Aboriginal owned and operated media, consultancy, and training organisations. This national campaign will consist of systematic advertising and promotion of iSISTAQUIT and video messages through various social media platforms that will be set-up, such as Facebook, Instagram, LinkedIn, and, Twitter. An Aboriginal owned company Ngakkan Nyaagu (NGNY) is developing an iSISTAQUIT website. The primary focus for distribution of the social media campaign will be through the early adopter service states namely Queensland, New South Wales, and South Australia. The ongoing management of the website and social media accounts will be led by Southern Cross University and will be updated regularly with advertising content such as professionally produced videos, photos, and posts.

*Video production*. Gilimbaa as the creative agency will conduct Community consultations (group discussions separately with Aboriginal and Torres Strait Islander women, health professionals and community leaders) to inform the creative approach and video content. Subject to pandemic and public health restrictions, it is anticipated that a series of short videos will be produced either in-house or in different locations across Australia, whichever is feasible. Key messaging in the videos will promote smoking cessation among Aboriginal and Torres Strait Islander women and families and highlight the importance of smoking cessation care, i.e., helping people and health practitioners feel confident and that it is worthwhile spending the time assisting pregnant women to quit. The videos will encourage Aboriginal and Torres Strait Islander women to seek and accept help from health providers, community, and family to quit smoking. A central Aboriginal talent will be engaged to present the iSISTAQUIT messages through short videos and bring to light community stories. Contingency plans will be made if the filming is interrupted due to travel restrictions.

### Outcome measures for iSISTAQUIT

#### Outcomes related to RE-AIM framework

This study will utilise the RE-AIM framework (Reach, Effectiveness, Adoption, Implementation and Maintenance) to plan, evaluate and report the intervention impact of the implementation of iSISTAQUIT (See Table 2). RE-AIM framework was chosen as it is a widely used implementation model that reports comprehensively on the key issues related to implementation studies- beginning with adoption and reach, followed by implementation and efficacy or effectiveness, and finishing with maintenance [46].

**Table 2:** RE-AIM framework evaluation.

RE-AIM component	Outcomes	Method of assessment (data sources)	Data sources
Reach	1. Service participation rate	• Number of HS participating in Evaluation-Research package / Number of HS approached (CL, RF) • Number of HS participating in Training only package/ number of HS approached (PTA, CCI)	• PenCS Topbar application or web-based version for iSISTAQUIT (ABCD template) or • Communicare iSISTAQUIT Clinical item (ABCD template)
	2. HCP participation rate	Number of HCP registered to do the training/ number of HCPs determined to be eligible to participate by the service (OM, RF)	
	3. Representativeness	Characteristics of HCPs such as age, sex and region of practice compared with national/regional figures available through national databases and peak bodies data (such as the General Practice Workforce providing Primary Care services in Australia)	Australian Government DOH General Practice Workforce providing Primary care services in Australia (https://hwd.health.gov.au/resources/data/gp-primarycare.html)
Effectiveness	1. Barriers and enablers	A 9 item, 5-point Likert scale (strongly disagree to strongly agree) questionnaire based on the Theoretical Domain Framework [49]. (OS)	• Online (through Moodle) pre-, immediate post- training and 3-month surveys
	2. Knowledge attitudes and practices related to SCC	Knowledge, Attitudes and Practices related to SCC measured through online pre-, immediate post- and 3 months post-training surveys. (OS)	• Online (through Moodle) pre-, immediate post- training and 3-month surveys
	3. Secondary outcomes	1. Smoking cessation rate: Percentage of women who change their smoking status (PTA, CCI) 2. Pregnant women participation rate: • Number of pregnant women who receive any iSISTAQUIT ABCD component/ number of eligible women seen by the service (PTA, CCI) • Number of pregnant women who receive My Journey booklet/ number of eligible women seen by the service (PTA,CL)	• PenCS Topbar application or web-based version for iSISTAQUIT (ABCD template) • Communicare iSISTAQUIT Clinical item (ABCD template) • Communicare iSISTAQUIT (smoking status) The ABCD tool includes a question that explores if the pregnant women quit smoking for at least 24 hours during the last 2 weeks, this question if available in the electronic medical records (PenCS Topbar application or web-based version for iSISTAQUIT or the Communicare iSISTAQUIT Clinical item). Additionally, a specific question regarding to the smoking status ‘do you smoke’ will be asked and recorded in Communicare. For all pregnant women that reported that they smoke, the status will be followed up and a percentage of the women that change of status reported by service.
Adoption	1. Practitioner participation rate	• Number of HCP who completed the training/ Number of HCPs who commenced the training (OM) (three separate rates calculated for HCPs who applied for CPD points, did not apply for CPD points and combined irrespective of CPD points). Rates will be compared statistically to determine if it’s worthwhile offering CPD points to improve uptake of training.	• Online (through Moodle, HPMI)
	2. Component adoption rate	• Four separate rates will be calculated for consultations in which A, B, C or D sections of the ABCD template were completed. This will be calculated by dividing number of respective completions (A, B, C or D) divided by number of consultations prompted by the app. The average component rate will be estimated for each component as the sum of the rates from each service divided by the number of participating services (PTA, CCI)	• PenCS Topbar application or web-based version for iSISTAQUIT (ABCD template) • Communicare iSISTAQUIT Clinical item (ABCD template)
Implementation	1. HCP iSISTAQUIT usage rate	• Number of HCP who used the iSISTAQUIT/ABCD template to engage women in smoking cessation care over the first 3 months /Number of HCP that commenced the training (OM, PTA, CCI) • Analysis of number and type of BCTs used from the template	• Online through Moodle, HPMI at 3 months and 6 months
Maintenance	1. Descriptive statistics (number and percentage)	• HCP’s/services that ask for more iSISTAQUIT resources (treatment manuals, patient flip charts, my journey booklets). (OS, AU) • HCPs that report iSQ training is a current part of their normal work. (OS) • HCPs that report iSQ training will become part of their normal work at 3 months post training. (OS) • Ongoing/long term use of the iSISTAQUIT ABCD template. (PTA, CCI) • Services interested in offering iSISTAQUIT training and resources (if available) to new HCPs joining the service after project completion. (QA)	• Qualitative analysis/audit of qualitative interviews by an external Aboriginal service. • The 3-month HCP survey. • Pen CS Topbar or Communicare de-identified aggregated quarterly data will provide iSISTAQUIT/ABCD template utilisation data until end of current study completion (31 December 2022). • End of study interviews

HS: health services; HCP: health care professional; ABS: Australian Bureau of Statistics.

CPD: Continued professional development; HPMI: Hunter Postgraduate Medical Institute; NRT: Nicotine Replacement therapy; iSQ: iSISTAQUIT

CL: Communication Log

RF: Registration form

RACGP: Royal Australian College of General Practitioners

PTA: Pen CS Topbar application

SCC: Smoking Cessation Care.

CCI: Communicare iSISTAQUIT Clinical Item

OS: Online Survey

QA: Quantitative analysis

AU: Audit

OM: Online through Moodle

#### Implementation readiness

When engaging the health services, interviews and questionnaires with CEOs, managers, and/or champions will be performed to evaluate the service readiness to implement the intervention. We will evaluate the system, organisational and staff capacity, functional considerations, the culture/climate, senior leadership, implementation plan and training. This baseline evaluation will allow us to identify the stage of the service and assist with the pre‐implementation and preparation. Participants will be invited to an interview and survey. The interview will be conducted over zoom, and the interviewer will share the information of the survey on the screen. The aim of the interview is to collect detailed information about the characteristics of the service before the implementation, and with the survey, we aim to assess the readiness to implement using the “Checklist to Assess Organisational Readiness (CARI) for Evidence Informed Practices Implementation” tool [47].

After concluding with the training, health care providers participating on the research arm will be invited to complete a survey using the NoMAD instrument [48] to assess the implementation process. The aim of this instrument is to include the perspective of individuals involved in the implementation work.

#### Research impact

For assessing research impact, we will use the Framework to Assess the Impact from Translational health research (FAIT). This framework includes metrics and a program logit structure. This tool is based on a modified program logic model that includes evaluation process, outputs, measurements of impact and process metrics (including research activities and research translation).

Additionally, end of study interviews will be conducted with Managers, Champions, and Health Care Practitioners. The interview guide will be designed based on the COM-B model.

#### Micro-costing analysis

A prospective micro-costing analysis of the implementation of the Evaluation-Research package (Option 1) will be conducted, including the design cost, initiation, and maintenance of the project. To estimate the cost, we will include the following information:

- Design of the intervention and its promotion cost that includes the cost of the design of the e-Learning tool
- Feedback from experts: Estimation of cost of meetings with different panels of experts
- iSISTAQUIT Staff costs: Cost of recruitment, training, implementing the intervention and follow up
- Health service costs: cost of appointments (number and duration of them), cost of training estimated by the duration of the training, cost of follow up meetings with the iSISTAQUIT team, among others.
- Intervention: material for the intervention (cost of time spent on the eLearning training printed material, eLearning design costs and website platform, NRT cost, smokerlyzers, etc)

#### Outcome measures related to social media campaign

Engagement, awareness and effectiveness of the social media campaign will be measured through a combination of methods including assessment of social media metrics of online content released by the campaign as well as surveys and qualitative interviews with stakeholders including health care providers, Aboriginal and Torres Strait Islander women and community members. More details are available from Table 3.

**Table 3:** Evaluation of social media campaign.

Characteristic	Details
**Target population**	Aboriginal and Torres Strait Islander audience exposed to the social media campaign including pregnant women, other women of reproductive age, their family members, their social contacts, and health care providers catering to Aboriginal women in pregnancy.
**Study design**	Survey, post-survey interview/focus group and social media surveillance
**Recruitment**	Aboriginal community participants will be recruited via social media (through active calls for participation via iSISTAQUIT and collaborators’, panel members’ and supporters’ websites and social media pages), participant interception at iSISTAQUIT study sites and community events. Participants will be asked to share the survey with others, or recommend them to take the survey, through snowball recruitment.
**Sample size**	If 50% of participants have heard of the campaign in the post period, a sample of 100 women from the iSISTAQUIT sites will enable estimation of this proportion with a 10% margin of error. Sample size for qualitative interviews will depend on numbers needed to attain data saturation.
**Data collection and outcome measures**	**Surveys:** To assess engagement, awareness and effectiveness of the social media campaign and related branding in influencing knowledge, attitudes and beliefs of pregnant women who smoke. **Qualitative interviews or focus groups:** conducted after about 3–6 months of campaign initiation with women, community members and Health Providers. A semi-structured interview schedule will be used to steer the interview/focus group discussion. Below are some examples of the questions included in the schedule: • What did you like about the campaign? What are we doing well? What should we continue to do? • What are we doing okay or badly with the campaign? • What we could have done instead? • Do you have any likes/dislikes / suggestions about the videos? • Have the videos changed your views or behaviour in any way? • Did you learn anything new? **Both surveys and interviews will be conducted together via personal intercept at the study sites.** **Social media metrics:** Social media metrics will be directly tracked and recorded from the social media webpages using platform specific social media tracking. IndigenousX will deliver these metrics in a detailed report. This will demonstrate the effectiveness of the campaign with the intended audience and the public. Google analytics will be used to profile the characteristics of the audience. Following metrics will be summarised both overall and by month: **Engagement**: Counts of retweets, likes, comments, link clicks, mentions, and direct messages received. **Content sharing**: Counts of testimonials, shares, reviews, guest posts, shared pictures at monthly intervals throughout the campaign **Audience characteristics**: Counts of total followers, new followers. This will also be broken down by users’ sex and age and location (Google analytics). **Content utilisation:** a) analysing comment sentiments (positive, neutral, or negative as supplied by IndigenousX), b) summary statistics of website utilisation will be presented, including the number of website sessions, total page views, bounce rate (single-page sessions divided by the number of total sessions on the website), pages per session, average session duration, mobile vs. desktop traffic.
**Analyses**	***Surveys*:** Surveys will be analysed descriptively. ***Social Media Metrics*:** Descriptive and summary statistics will be used to analyse trends in social media metrics. Regression analysis will be used determine the relationship between the user characteristics (e.g., age, sex, region etc.) and social media metrics. ANOVA/t-tests or their non-parametric counterparts will be used to assess if there are statistically significant differences in user engagement (e.g., number of likes, shares etc.) across different social media platforms. We will conduct a qualitative sentiment analysis of textual content (comments, tweets, post from users and direct messages) posted on the social media pages to classify the emotions expressed by the users in these posts. Textual content will also be analysed using thematic analysis to identify predominant themes, which will then be used to tailor the content posted on the social media and iSISTAQUIT website to improve user engagement and useability of the webpages. **Qualitative interviews/ focus groups:** Focus groups and interviews will be audio-recorded and transcribed followed by thematic analysis.

### Data collection

All those who participate in the Evaluation-Research package (Option 1), data will be collected by on-line survey links for HCPs, the supplied iSISTAQUIT practice software templates and routinely collected data from services’ databases (clinical information systems). The health professionals enter the data about the patient’s characteristics and information about their quit attempt using the supplied on-line template, via the Pen CS iSISTAQUIT Topbar app or web-link version or via the Communicare iSISTAQUIT clinical item. Quarterly aggregated data will be created within Microsoft PowerBI for the Pen Cs Topbar App template. Services that do not have access to electronic clinical information systems utilising Pen CS Topbar app or Communicare template will be provided with an alternate method to record the data from the iSISTAQUIT template during the consultation with the patient. The quarterly aggregated data report will then be generated by the health service using R/RStudio.

At the end of the study, qualitative interview data with health practitioners, managers, and champions will be collected and analysed by an external research agency to prevent bias.

#### Evaluation plan

iSISTAQUIT training: Evaluation will be done using the RE-AIM framework, which systematically evaluates the Reach, Effectiveness, Adoption, Implementation and Maintenance of implementation studies. For details see, Table 2.

Descriptive statistics will be used to present numbers, rates, proportions and trends in the data collected through surveys, Likert scales and service level data available through data collection tools described in Table 2. Thematic analysis will be used to analyse qualitative data collected during end of study interviews with practitioners, managers and research facilitators at the services.

Knowledge, attitudes and practices related to SCC: Differences in practitioner online survey responses from pre-training (referent period) to immediate post- and 3 months post-training will be assessed using linear mixed effects regression models, including fixed categorical effects for time, and a random intercept for site to model correlated responses for practitioners from the same service.

Social media campaign: The success of social media campaign will be assessed using surveys, qualitative methods, and social media metrics. More details are available from Table 3.

### Dissemination

The results of the evaluation will be disseminated to the stakeholders, Aboriginal and Torres Strait Islander and non-Aboriginal and Torres Strait Islander community members and wider national and international research fraternity using a range of academic and non-academic dissemination channels, namely scientific journals, conferences, community talks, email newsletters and social media. Academic channels will include presentation of findings at academic conferences (local, national, and international) as well as publication in open access journals to facilitate greater access to researchers and stakeholders in resource poor settings. Non-academic dissemination will include circulation of an individualized progress report/newsletter to all participating services and organisations. If COVID-19 impact continues, all in-person presentations will be replaced by Zoom presentations at sites where travel restrictions are in place. Results will also be disseminated to the stakeholders and public via the iSISTAQUIT website and social media pages using engaging content to further improve user engagement with iSISTAQUIT. A policy report including recommendations will be written and disseminated to peak bodies and stakeholder organisations.

### Governance

The iSISTAQUIT project is governed by an academic panel comprising Aboriginal and Torres Strait Islander people, Indigenous people from other nations, and non-Indigenous people. A National Aboriginal and Torres Strait Islander Advisory Panel provides cultural oversight. A Social Media Panel provides guidance on the social media campaign; an educational panel guides the e-Learning modules and the medical practice software development. Membership of the panels is from peak organisations and health services and comprises at least 50% Aboriginal and Torres Strait Islander people. A recruitment and engagement framework is being co-developed with Aboriginal stakeholders and panel members to provide the cultural direction and a values base for the research and all its aspects. The recruitment and engagement framework describes how the iSISTAQUIT project is guided by peak bodies, Aboriginal health services and community members. Our belief is working together in common goals to support communities to live their healthiest life by quitting smoking. iSISTAQUIT values two-way shared learning and upskilling health care professionals for long term sustainability. Connection via referral pathways, promotion and partnerships is key. The co-developed engagement values are core to the project.

#### Status and timeline

The recruitment of sites has begun and to date 92 health providers have been enrolled and 42 health providers have completed training. Site recruitment will be completed by August 2022. All participating HCPs will complete training by October 2022. Data collection will be completed by November 2022. Data analysis and reporting of results will be completed by December 2022. The social media campaign was launched in December 2021 and evaluation completed by October 2022.

## Conclusion

Smoking in pregnancy is a very high priority for Aboriginal and Torres Strait Islander peoples nationwide. Although numerous community-led and local initiatives are underway within many Aboriginal communities in Australia, there is no effective national smoking cessation program for Aboriginal women. iSISTAQUIT program would facilitate rapid translation of research into practice.

This innovative research, using a multi-component intervention, aims to practically apply and integrate what is already known into a highly translatable approach in real-world primary care settings into AMS and Mainstream services. The results from this study will develop new knowledge about digitally delivered smoking cessation training for health professionals who care for pregnant Aboriginal and Torres Strait Islander women. The results from this study will also inform the GACD/NHMRC funded iSISTAQUIT scale-up project (reference: GNT2009206).
